# Insufficient sensitivity of joint aspiration during the two-stage exchange of the hip with spacers

**DOI:** 10.1186/s13018-017-0703-z

**Published:** 2018-01-10

**Authors:** Sebastian Philipp Boelch, Manuel Weissenberger, Frederik Spohn, Maximilian Rudert, Martin Luedemann

**Affiliations:** Department of Orthopaedic Surgery, Julius-Maximilians University Wuerzburg, Koenig-Ludwig-Haus, 11 Brettreichstrasse, 97074 Wuerzburg, Germany

**Keywords:** Two-stage exchange, Hip, Periprosthetic infection, Joint aspiration, Spacer

## Abstract

**Background:**

Evaluation of infection persistence during the two-stage exchange of the hip is challenging. Joint aspiration before reconstruction is supposed to rule out infection persistence. Sensitivity and specificity of synovial fluid culture and synovial leucocyte count for detecting infection persistence during the two-stage exchange of the hip were evaluated.

**Methods:**

Ninety-two aspirations before planned joint reconstruction during the two-stage exchange with spacers of the hip were retrospectively analyzed.

**Results:**

The sensitivity and specificity of synovial fluid culture was 4.6 and 94.3%. The sensitivity and specificity of synovial leucocyte count at a cut-off value of 2000 cells/μl was 25.0 and 96.9%. C-reactive protein (CRP) and erythrocyte sedimentation rate (ESR) values were significantly higher before prosthesis removal and reconstruction or spacer exchange (*p* = 0.00; *p* = 0.013 and *p* = 0.039; *p* = 0.002) in the infection persistence group. Receiver operating characteristic area under the curve values before prosthesis removal and reconstruction or spacer exchange for ESR were lower (0.516 and 0.635) than for CRP (0.720 and 0.671).

**Conclusions:**

Synovial fluid culture and leucocyte count cannot rule out infection persistence during the two-stage exchange of the hip.

## Background

Total hip arthroplasty is regarded as one of the most successful surgeries of the past century, and numbers are consistently increasing. At the same time, the absolute number of two-stage revisions is increasing, too [1]. Periprosthetic joint infection (PJI) of the hip occurs in about 1% of primary total hip arthroplasties. However, it has become one of the main reasons for arthroplasty revision [2]. PJI is a potentially devastating complication and can lead to a high level of morbidity and mortality [3]. Additionally, costs for PJI treatment are expected to exceed 1.6 billion dollars in the USA by 2020 [4]. Although the single-stage exchange is on the rise, the gold standard for the treatment of chronic PJI remains the two-stage exchange [2, 3, 5].

During the stage one operation, the infected prosthesis is removed (prosthesis removal (PR)) and the infected tissue is debrided. To the surgeons’ preference, either an articulating spacer or a girdlestone spacer is implanted or a girdlestone situation is created. PR is followed by a course of antibiotics. Then, depending on evaluation of infection eradication, the joint is reconstructed or the spacer is exchanged (reconstruction or spacer exchange (ROS)). Whether or not to perform the two-stage exchange with or without a break of systemic antibiotic administration to evaluate infection eradication has been controversial for over 30 years [6]. Kapadia et al. and Sandiford et al. assessed evaluation of infection eradication after such a drug holiday as the standard method. In this setting, joint aspiration before ROS (interstage aspiration (IA)) of the hip is regarded as an extended diagnostic procedure to verify infection eradication [2, 3]. Joint aspiration is a major pillar for the diagnosis of PJI [7–9]. At the hip, sensitivity of synovial fluid culture (SFC) has shown a wide range between 44 and 80%. Sensitivity of synovial leucocyte count (SLC) seems to be higher with 82 to 96% [10–12]. However, although data is very limited, the diagnostic value of IA seems to be worse [13]. Currently, suggestions to abandon the drug holiday towards a continuous antibiotic administration are discussed, warranted by a shorter spacer interval and therefore better quality of life and lower comorbidity [5]. Consecutively, IA with culture for diagnosis of infection persistence would not be reasonable anymore.

With this retrospective study of 92 cases, we present our results of SFC and SLC at IA with an indwelling spacer, as well as the value of the C-reactive protein (CRP), erythrocyte sedimentation rate (ESR), and serum white blood cell count (WBC).

## Methods

After the approval by the institution’s ethics review board, the electronic database of our orthopedic department was retrospectively scanned for all hip arthroplasty exchanges between 20 December 2008 and 9 June 2016 yielding 949 cases. All cases of two- or more-stage joint exchange for the treatment of PJI of the hip that had undergone IA before ROS (*N* = 102) were included. Then, one case without a result of SFC, 6 cases with a drug holiday shorter than 7 days, and 3 cases that were treated via a temporary girdlestone situation were excluded. This resulted in 92 cases for statistical analysis. PJI was diagnosed according to the Clinical Practice Guidelines by the Infectious Disease Society of America [8].

The mean duration from index surgery to PR was 58.75 months (1175 days (2–8338)) with a median of 14.38 months (431.5 days). 47.8% of the PRs were primary revisions. 57.6% were male patients. The mean age at PR was 67.46 years (40–88) and the mean BMI 29.8 kg/cm^2^ (19.3–52.6). At the preference of the surgeon, an articulating (91.3%) or a girdlestone spacer (8.7%) was implanted. Spacers were molded by hand with a Steinman pin as an endoskeleton. Palacos R+G with addition of 2 g of vancomycin per 40 cc batch was routinely used. If preoperative cultures from aspiration yielded no growth, the antibiotic therapy was started with a combination of an aminoglycoside and a cephalosporin. In case of bacteria detection, antibiotic therapy was adapted according to the microbiologist’s recommendation. The mean duration of intravenous antibiotic administration was 18.5 days (3–52) followed by a course of oral antibiotic therapy for a mean of 17.0 days (3–38). The mean combined duration of antibiotic therapy was 34.4 days (20–74). The mean drug holiday was 15.3 days (7–73). 72.8% of the IAs were performed after a drug holiday of at least 14 days. Aspiration was performed under sterile conditions, and intraarticular sampling was verified with fluoroscopy. Two to five milliliters of the aspirate were inoculated into blood culture bottles containing 32 ml of a complex Trypticase-Soja-Bouillon and 8 ml of a charcoal suspension (bac/Tec Alert FN (bioMérieux)) for transportation. With the remaining aspirate, SLC was performed. All microbial samples were cultivated for at least 14 days before ROS was performed. The mean duration between PR and ROS was 69.8 days (48–126) with joint reconstruction in 89 cases and 3 cases with a spacer exchange. A mean of 3 (2–5) microbiologic samples were taken at ROS. Additional tissue samples were collected for histopathological evaluation. Persistent infection was defined as (a) positive intraoperative cultures, (b) histologic signs of acute inflammation, (c) periprosthetic membranes classified as type II or III according to Krenn and Morawietz [14], and (d) intraoperatively evident pus at ROS. IA was considered true positive (tp) if SFC showed bacteria growth, or the SLC was ≥ 2000/μl in cases of persistent infection. In cases of negative SFC or a SLC < 2000/μl but persistent infection, IA was considered false negative (fn). Sensitivity was defined as tp/(tp + fn) and specificity as tn/(tn + fp). CRP, ESR, and WBC values of the infection persistence group and infection eradication group were compared with the Mann-Whitney *U* test before PR and before ROS. Level of significance was *p* < 0.05. As a quality measure, the receiver operating characteristic area under the curve (ROC AUC) was calculated. All statistics were conducted with SPSS version 23 (SPSS Inc. Chicago, IL, USA).

## Results

Infection was eradicated in 70 cases (76.1%). In 4 cases (4.3%), the same causative bacteria were found at PR and ROS. Of the 17 bacteria detected at ROS, 13 were different to bacteria detected at PR, giving a bacteria shift of 76.47% (Table 1).

**Table 1:** Bacteria detected at prosthesis removal and at joint reconstruction or spacer exchange

	Prosthesis removal			Joint reconstruction or spacer exchange
	Total *N* (%)	Infection persistence *N*	Infection eradication *N*	Infection persistence *N* (%)
Unimicrobial infections				
Staphylococcus epidermidis	14 (18.67)	4	10	12 (70.6)
Staphylococcus aureus	10 (13.33)	4	6	2 (11.7)
Enterococcus faecalis	9 (12.00)	1	8	–
Propionibacterium acnes	6 (8.00)	2	4	2 (11.7)
Other coagulase-negative Staphylococci	6 (8.00)	2	4	1 (5.9)
Propionibacterium spp.	4 (5.33)	1	3	–
Staphylococcus capitis	2 (2.67)	0	2	–
Streptococcus agalactiae	2 (2.67)	0	2	–
Corynebacterium spp.	2 (2.67)	0	2	–
Other single	7 (9.33)	2	5	–
Polymicrobial infections				
Propionibacterium acnes + other	5 (6.67)	0	5	–
Staph epi + other	5 (6.67)	2	3	–
Other	3 (4.00)	1	2	–
Bacteria detections’ total	75 (100)	19	56	17 (100)
Sterile	17	3	14	5

**Fig. 1:**
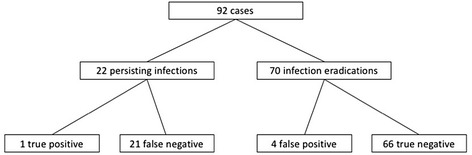
Flow chart of synovial fluid culture categorized by infection eradication and infection persistence: 92 cases of two-stage exchange with a spacer were analyzed. We found 22 cases with persistent infection, defined by intraoperative samples for culture and histopathologic examination or intraoperatively evident pus. In the group of infection persistence, one culture was positive and 21 were negative at interstage aspiration. In the group of 70 cases with infection eradication, 4 cultures yielded growth and 66 did not at interstage aspiration

SFC yielded a sensitivity of 4.6% and a specificity of 94.3% (Table 2) with one of 22 SFCs at IA that was true positive for infection persistence (Fig. 1). In this case, the SFC at IA showed the same bacteria as the tissue cultures at ROS. Of the four false positive SFCs in the infection eradication group, three were recognized as contamination before ROS with joint reconstruction. They yielded coagulase negative cocci once, Micrococcus species once, and Staphylococcus epidermidis once. In the last case of false positive SFC, which yielded Staphylococcus epidermidis, the spacer was exchanged.

**Table 2:** Results of interstage aspiration

	Sensitivity (%)	Specificity (%)	Positive predictive value (%)	Negative predictive value (%)
Synovial fluid culture	4.55	94.29	20	75.86
Synovial fluid leucocyte count	25.00	96.86	66.67	82.5

**Fig. 2:**
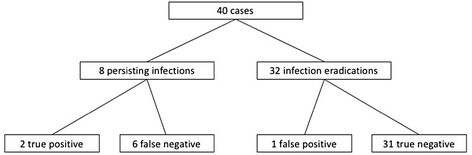
Flow chart of synovial fluid count categorized by infection eradication and infection persistence: 40 cases of two-stage exchange with a spacer were analyzed. We found 8 cases with persistent infection, defined by intraoperative samples for culture and histopathologic examination or intraoperatively evident pus. In the group of infection persistence, synovial leucocyte count was ≥ 2000/μl in 2 and < 2000/μl in 6 interstage aspirations. In the group of 32 cases with infection eradication, synovial leucocyte count was ≥ 2000/μl in one and < 2000/μl in 31 interstage aspirations

In 40 cases, synovial fluid could be sent for SLC at IA (Fig. 2). Of the 8 cases with persisting infection, 2 were true positive giving a sensitivity of 25.0% and specificity of 96.9% (Table 2).

**Fig. 3:**
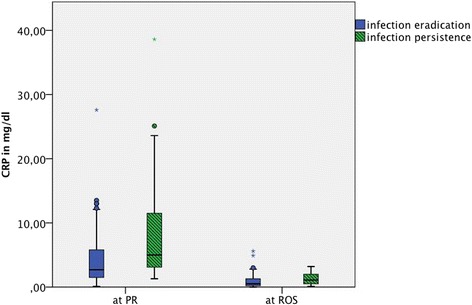
Box plots of CRP values before prosthesis removal (PR) and reconstruction or spacer exchange (ROS) categorized by infection eradication and infection persistence

CRP levels were significantly higher in the infection persistence group than in the infection eradication group at PR (*p* < 0.00). At ROS, the median CRP in the infection persistence group of 1.10 mg/dl (0.87–1.69) was also significantly higher (*p* = 0.013) than in the infection eradication group with a median CRP of 0.50 mg/dl (0.62–1.12) (Fig. 3). The ROC AUC at diagnosis was 0.720 and 0.671 before ROS. Similar results were found for the ESR (*p* = 0.039 at PR and 0.002 at ROS); however, ROC AUCs were lower (0.516 and 0.635). The serum WBC showed no significant differences.

## Discussion and conclusions

Joint aspiration plays a fundamental role in the diagnosis of PJI. While PJI causative bacteria identification is crucial in primary diagnosis, IA is supposed to rule out infection persistence. Under this aspect, our results question the relevance of IA during the two-stage exchange. The SFC had a very low sensitivity of only 4.6%. There are only few other studies investigating the value of IA. Hoell et al. found almost the same sensitivity in their study of 115 hip and knee periprosthetic joint infections [15]. In the only other study investigating IA for hip spacers, Newman et al. found a sensitivity of 30% [13]. These low sensitivities may be explained by high local antibiotic concentration due to the indwelling spacer. However, Janz et al. also demonstrated a low sensitivity of SFC of 13% in 69 girdlestone hips [16]. Additionally, we found a high bacteria shift rate of 76.5%. This rate compares to the rate of 75% observed by Hoell et al. This shift seems to tend towards potentially biofilm-forming bacteria as the rate of Staphylococcus epidermidis and aureus-associated infections increased from 42.7% at PR to 82.4% at ROS in our study. Thus, biofilm formation may be an explanation for the poor results of SFC. Accordingly, the indirect means of persistent infection detection such as SLC had a higher sensitivity, a fact known for primary PJI diagnosis [9, 10]. In comparison to the categorical SFC, the cut-off value of the metric SLC can be leveled up or down. It is evident that persistent infection can produce significantly higher SLC at IA [17]. However, the best cut-off values for SLC are still to be discussed [8, 18]. Irrespectively, whether IA was performed at the hip or at the knee Hoell et al. calculated the best sensitivity at a cut-off value of only 960 cells/μl with a sensitivity of 31.3%, but at the expense of a very low specificity of 39.1% [15]. Comparable low sensitivity and specificity for SLC was found by Zmistowski et al. [17]. However, derived from the data of primary PPI diagnosis, the different presuppositions at the knee in contrast to the hip in order to determine the best cut-off values should be considered [9, 12, 18]. At the hip, Newman et al. recently suggested a new SLC threshold of 1166 cells/μl with a sensitivity of 76% and specificity of 78% [13]. We chose a SLC cut-off value of 2000 cells/μl, which is one of the lowest recommended thresholds for primary PJI [9]. Compared to Newman et al., this threshold yielded a lower sensitivity of 25.0%. However, the high specificity of 96.9% reduces the risk of inadequate spacer exchanges instead of joint reconstructions. Thus, SLC could be used as an intraoperative tool at ROS to confirm infection persistence in cases with clinical suspicion. This is particularly underlined by the fact that there is a lack of other intraoperative possibilities. In this study, 5 cases of persistent infection were defined by the histopathologic tissue samples at ROS, while cultures remained sterile. However, a small study by Cho et al. could not demonstrate the impact of fresh frozen sections on the outcome after ROS [19]. Other means, such as the leucocyte esterase test or the alpha-defensin immunoassay, have not been investigated at the second stage of the two-stage exchange [20].

Besides the retrospective design, there are other limitations of this study. First, we could only analyze SLC in 40 cases. This was owed to the approach with first inoculating the blood culture bottles for SFC and then using the remaining aspirate for SLC. On the basis of the new data, SLC should be preferred to SFC, if IA is performed.

As a further limitation, the exact amount of aspirate volume for SFC could not be analyzed in this study. Renz et al. recommended pediatric blood culture bottles with 20 ml medium to reduce the required aspirate volume [9]. Under this aspect, ultrasound-guided aspiration may be advantageous in contrast to fluoroscopic guidance because the joint fluid volume is visualized before aspiration. Selecting patients with sufficient joint fluid volume might increase sensitivity for SFC. Additionally, ultrasound-guided aspiration is less expensive and does not afford radiation. As a disadvantage, it requires a trained person and sterility is an issue. At least in the setting of initial PPI diagnosis, the superiority of one of these approaches concerning sensitivity and specificity could not be demonstrated [21, 22].

So far, infection persistence is not consistently defined. Studies evaluating the success rates of the two-stage exchange consider repeated septic revision after joint reconstruction as treatment failure [2]. However, Fehring et al. could show that in 50% of repeated two-stage exchanges cultures yield other bacteria than at the previous two-stage exchange. Thus, reinfection after a two-stage exchange should not be regarded as the definition for insufficient infection eradication [23]. Newman et al. used a modification of the Musculoskeletal Infection Disease Society criteria for PPI to evaluate infection persistence before ROS. But with this approach, the investigated method is included in the reference evaluation [13]. We used the intraoperative tissue cultures and the histopathologic samples at ROS to define infection persistence. In 12 ROSs, Staphylococcus epidermidis was detected, a pathogen that is a commercial of the skin flora and thus might be a contaminant. However, in 8 cases, Staphylococcus epidermidis could be consecutively cultured in two or more intraoperative samples. In 3 cases with only one positive intraoperative culture complex resistance patterns against oxacillin, rifampicin or fusidin were tested, respectively. In these cases, histopathologic evaluation confirmed persistent infection. Because of controversial data during the two-stage exchange, we decided against the use of CRP and ESR for the definition of infection persistence. For example, Kusuma et al. found lower CRP values in the infection persistence group for PJI of the knee [24]. Additionally, Hoell et al. showed a low sensitivity for CRP of only 43% [15]. However, in retrospect, we found significantly higher CRP values before ROS. Interestingly, in the infection persistence group, CRP values were already significantly higher before PR, too.

27.2% of the IA were performed with a drug holiday of 7 to 14 days. These 25 cases included 4 cases of infection persistence. Of these, all SFCs were false negative. Two patients were off antibiotics for 10 and the other two for 7 days. If the SFCs of these patients had been true positive, the sensitivity of SFC would have increased by 14.4%. Thus, if IA is preformed, the 14-day drug holiday should be respected carefully. Because of the small number of patients in this study, additional factors that may influence IA results, such as chronic inflammatory diseases or immunomodulation, need to be investigated in future studies.

Due to the lack of a reliable diagnostic tool to rule out infection persistence, every indication for joint reconstruction must be evaluated individually.

Summarizing our results, neither SFC nor SLC at IA during the two-stage exchange of the hip with a spacer is reliable as a standard approach for ruling out infection persistence. Thus, we recommend against the routine performance of IA during the two-stage exchange at the hip. Instead, ROS should be performed without cessation of the systemic antibiotic therapy. A high CRP before PR and ROS must be considered a hint towards increased risk of infection persistence. In these cases and in cases of clinical signs of infection persistence, SLC should be performed at ROS to confirm infection persistence.

## Abbreviations

CRP: C-reactive protein
ESR: Erythrocyte sedimentation rate
fn: False negative
fp: False positive
IA: Interstage aspiration
PJI: Periprosthetic joint infection
PR: Prosthesis removal
ROC AUC: Receiver operating characteristic area under the curve
ROS: Reconstruction or spacer exchange
SFC: Synovial fluid culture
SLC: Synovial leucocyte count
tn: True negative
tp: True positive
WBC: Serum white blood cell count
